# [(*Z*)-1-Amino-2-cyano-2-(4,6-di-2-pyridyl­pyrimidin-2-yl)ethenolato]chlorido(*N*,*N*-dimethyl­formamide-κ*O*)zinc(II)

**DOI:** 10.1107/S1600536810017770

**Published:** 2010-05-22

**Authors:** Fangfang Jian, Mei Du

**Affiliations:** a New Materials & Function Coordination Chemistry Laboratory, Qingdao University of Science & Technology, Qingdao 266042, People’s Republic of China

## Abstract

In the title complex, [Zn(C_17_H_11_N_6_O)Cl(C_3_H_7_NO)], the Zn^II^ atom has a distorted square-pyramidal coordination formed by one Cl, two O and two N atoms. In the crystal structure, inter­molecular N—H⋯Cl hydrogen bonds link mol­ecules into centrosymmetric dimers, which are further assembled by π–π inter­actions [centroid–centroid distances = 3.809 (3) and 3.834 (3) Å] into layers parallel to the *ab* plane. The crystal packing exhibits also weak inter­molecular C—H⋯Cl inter­actions.

## Related literature

For general background concerning the self-assembly of metal complexes with organic ligands, see: Chi *et al.* (2008 ▶); Patroniak *et al.* (2005 ▶); Kovbasyuk *et al.* (2005 ▶). For related structures, see: Preston & Kennard (1969 ▶); Jian *et al.* (2004 ▶); Patroniak *et al.* (2003 ▶). 
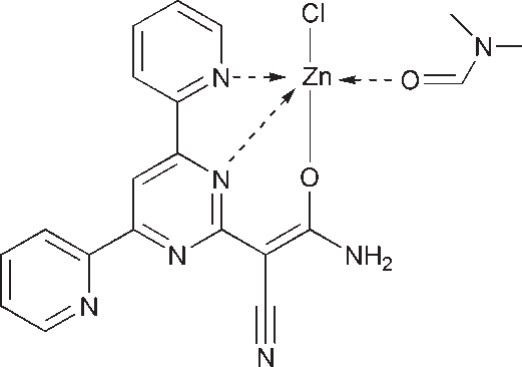


## Experimental

### 

#### Crystal data

[Zn(C_17_H_11_N_6_O)Cl(C_3_H_7_NO)]
*M* *_r_* = 489.23
Triclinic, 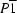

*a* = 8.5610 (17) Å
*b* = 11.250 (2) Å
*c* = 12.259 (3) Å
α = 110.70 (3)°
β = 91.36 (3)°
γ = 101.37 (3)°
*V* = 1077.1 (4) Å^3^
*Z* = 2
Mo *K*α radiation
μ = 1.30 mm^−1^
*T* = 295 K
0.20 × 0.17 × 0.15 mm

#### Data collection

Enraf–Nonius CAD-4 diffractometer
10564 measured reflections
4863 independent reflections
4214 reflections with *I* > 2σ(*I*)
*R*_int_ = 0.026
3 standard reflections every 100 reflections  intensity decay: none

#### Refinement

*R*[*F* ^2^ > 2σ(*F* ^2^)] = 0.049
*wR*(*F* ^2^) = 0.167
*S* = 1.09
4863 reflections
284 parameters
H atoms treated by a mixture of independent and constrained refinement
Δρ_max_ = 1.58 e Å^−3^
Δρ_min_ = −0.58 e Å^−3^

### 

Data collection: *CAD-4 Software* (Enraf–Nonius, 1989 ▶); cell refinement: *CAD-4 Software*; data reduction: *NRCVAX* (Gabe *et al.*, 1989 ▶); program(s) used to solve structure: *SHELXS97* (Sheldrick, 2008 ▶); program(s) used to refine structure: *SHELXL97* (Sheldrick, 2008 ▶); molecular graphics: *SHELXTL* (Sheldrick, 2008 ▶); software used to prepare material for publication: *WinGX* (Farrugia, 1999 ▶).

## Supplementary Material

Crystal structure: contains datablocks global, I. DOI: 10.1107/S1600536810017770/cv2708sup1.cif

Structure factors: contains datablocks I. DOI: 10.1107/S1600536810017770/cv2708Isup2.hkl

Additional supplementary materials:  crystallographic information; 3D view; checkCIF report

## Figures and Tables

**Table 1:** Hydrogen-bond geometry (Å, °)

*D*—H⋯*A*	*D*—H	H⋯*A*	*D*⋯*A*	*D*—H⋯*A*
N6—H6*A*⋯Cl1^i^	0.86	2.57	3.396 (4)	161
C10—H10*A*⋯Cl1^ii^	0.93	2.80	3.670 (4)	156

Symmetry codes: (i) 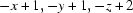; (ii) 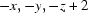.

**Fig. 1.**
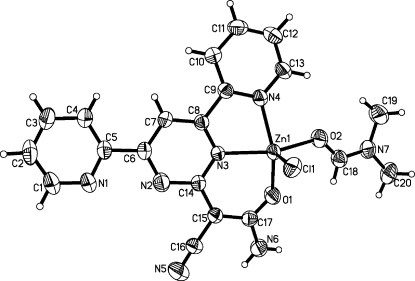
The structure of the title compound showing 50% probability displacement ellipsoids and the atom-numbering scheme.

## supplementary crystallographic information

### Comment

Recently, self-assembly of specially designed metal complexes have attracted intense attention due to their fascinating molecular structures and crystal-packing motifs (Chi *et al.*, 2008). Several metal complexes with ligands containig heterocyclic pyrimidine and pyridine units have been studied previously  to explore the role of hydrogen bonding in supramolecular assemblies (Patroniak *et al.*, 2005; Kovbasyuk *et al.*, 2005). Herein, we report the crystal structure of the title compound (I).

In (I) (Fig. 1), the zinc(II) ion is coordinated by one O anion and two N atoms of (*Z*)-3-amino-2-(4,6-di(pyridin-2-yl)pyrimidin-2-yl)-3- hydroxyacrylonitrile ligand, one O atom from DMF (DMF = *N*,*N*-dimethylformamide) and one Cl^-^ anion, forming a distorted square-pyramidal geometry. The Zn—Cl distance of 2.2956 (12)Å is longer than the value of 2.212 (4) Å in ZnCl_2_(2,9-dimethyl-1,10-phenanthroline) (Preston *et al.*, 1969) and 2.209 (1)Å in ZnCl_2_(C_6_H_4_N_3_CH_2_COPh)_2_ (Jian *et al.*, 2004). The Zn—O bond lengths of 1.996 (3) and 2.066 (2)Å, respectively, are shorter than those reported previously (Patroniak *et al.*, 2003). The Zn—N bond lengths are 2.103 (3) and 2.106 (2) Å, respectively, which are in disagreement with the corresponding five-coordination Zn—N distances found in similar compounds (Patroniak *et al.*, 2003; Chi *et al.*, 2008 ). The N3—Zn1—N4 bite angle is 77.58 (10)°, which is narrower than that in {Zn[4,6-bis(2-pyridyl)-2-aminopyrimidine](OOCCH_3_)_2_} [78.40 (7)°, Chi *et al.*, 2008].

The five-membered chelate ring N3/C8/C9/N4/Zn1 (P1) is fairly planar, the deviation of atom Zn1 from the weighted least-squares plane N3/C8/C9/N4 is 0.079 Å. The dihedral angles formed by P1 with the planes N3/C14/C15/C17/O1/Zn1(P2), N2/C6—C8/N3/C14(P3), N4/C9—C13(P4) and N1/C1—C5(P5) are 16.67, 9.93, 5.02 and 19.49°, respectively. The dihedral angles formed by P3 with P4 and P5 are 5.61 and 16.55°, respectively. The dihedral angle between P2 and P4 is 14.55°.

In the crystal structure, intermolecular N—H···Cl hydrogen bonds (Table 2) link molecules into centrosymmetric dimers, which are further assembled by π-π interactions (Table 1) into layers parallel to *ab* plane. The crystal packing exhibits also weak intermolecular C—H···Cl interactions (Table 2).

### Experimental

The title complex was prepared by the reaction of (*Z*)-3-amino-2-(4,6-di(pyridin-2-yl)pyrimidin-2-yl)-3-\ hydroxyacrylonitrile (3.16 g, 10 mmol) with Zinc dichloride (1.36 g, 10 mmol) in water solution at 353 K for four hours. Single crystals suitable for x-ray measurements were obtained by recrystallization from DMF at room temperature.

### Refinement

All H atoms, except H1, were fixed geometrically and allowed to ride on their attached atoms, with C—H distances constrained to 0.93-0.96Å, N—H = 0.86 Å, and with U_iso_(H) = 1.2-1.5U_eq_ (C, N). Atom H1 was located on a difference map and refined isotropically.

### Crystal data

**Table:**

[Zn(C_17_H_11_N_6_O)Cl(C_3_H_7_NO)]	*Z* = 2
*M**_r_* = 489.23	*F*(000) = 500
Triclinic, *P*1	*D*_x_ = 1.509 Mg m^−^^3^
Hall symbol: -p 1	Mo *K*α radiation, λ = 0.71073 Å
*a* = 8.5610 (17) Å	Cell parameters from 25 reflections
*b* = 11.250 (2) Å	θ = 4–14°
*c* = 12.259 (3) Å	µ = 1.30 mm^−^^1^
α = 110.70 (3)°	*T* = 295 K
β = 91.36 (3)°	Block, red
γ = 101.37 (3)°	0.20 × 0.17 × 0.15 mm
*V* = 1077.1 (4) Å^3^	

### Data collection

**Table:**

Enraf–Nonius CAD-4 diffractometer	*R*_int_ = 0.026
Radiation source: fine-focus sealed tube	θ_max_ = 27.5°, θ_min_ = 3.0°
graphite	*h* = −10→11
ω scans	*k* = −14→14
10564 measured reflections	*l* = −15→15
4863 independent reflections	3 standard reflections every 100 reflections
4214 reflections with *I* > 2σ(*I*)	intensity decay: none

### Refinement

**Table:**

Refinement on *F*^2^	Primary atom site location: structure-invariant direct methods
Least-squares matrix: full	Secondary atom site location: difference Fourier map
*R*[*F*^2^ > 2σ(*F*^2^)] = 0.049	Hydrogen site location: inferred from neighbouring sites
*wR*(*F*^2^) = 0.167	H atoms treated by a mixture of independent and constrained refinement
*S* = 1.09	*w* = 1/[σ^2^(*F*_o_^2^) + (0.1038*P*)^2^ + 0.732*P*] where *P* = (*F*_o_^2^ + 2*F*_c_^2^)/3
4863 reflections	(Δ/σ)_max_ = 0.001
284 parameters	Δρ_max_ = 1.58 e Å^−^^3^
0 restraints	Δρ_min_ = −0.58 e Å^−^^3^

### Special details

**Table:**

Geometry. All esds (except the esd in the dihedral angle between two l.s. planes) are estimated using the full covariance matrix. The cell esds are taken into account individually in the estimation of esds in distances, angles and torsion angles; correlations between esds in cell parameters are only used when they are defined by crystal symmetry. An approximate (isotropic) treatment of cell esds is used for estimating esds involving l.s. planes.
Refinement. Refinement of *F*^2^ against ALL reflections. The weighted *R*-factor wR and goodness of fit *S* are based on *F*^2^, conventional *R*-factors *R* are based on *F*, with *F* set to zero for negative *F*^2^. The threshold expression of *F*^2^ > σ(*F*^2^) is used only for calculating *R*-factors(gt) etc. and is not relevant to the choice of reflections for refinement. *R*-factors based on *F*^2^ are statistically about twice as large as those based on *F*, and *R*- factors based on ALL data will be even larger.

### Fractional atomic coordinates and isotropic or equivalent isotropic displacement parameters (Å^2^)

**Table:**

	*x*	*y*	*z*	*U*_iso_*/*U*_eq_	
Zn1	0.33598 (4)	0.22665 (3)	0.92885 (3)	0.03870 (16)	
Cl1	0.17317 (10)	0.36222 (8)	1.02298 (8)	0.0497 (2)	
O1	0.5618 (3)	0.3196 (3)	0.9911 (2)	0.0511 (6)	
O2	0.3716 (3)	0.2649 (3)	0.7773 (2)	0.0542 (6)	
N1	0.3389 (4)	−0.0424 (3)	1.3640 (3)	0.0566 (8)	
N2	0.4266 (4)	0.0756 (3)	1.2026 (3)	0.0496 (7)	
N3	0.3565 (3)	0.1094 (2)	1.0273 (2)	0.0328 (5)	
N4	0.1814 (3)	0.0487 (3)	0.8275 (2)	0.0401 (6)	
N5	0.7321 (5)	0.3459 (4)	1.3637 (3)	0.0727 (10)	
N6	0.7680 (4)	0.4302 (3)	1.1244 (3)	0.0591 (8)	
H6A	0.8020	0.4736	1.0811	0.071*	
H6B	0.8209	0.4462	1.1904	0.071*	
N7	0.4824 (4)	0.3814 (3)	0.6721 (3)	0.0499 (7)	
C1	0.3016 (6)	−0.0996 (5)	1.4418 (4)	0.0646 (11)	
H1B	0.3465	−0.0551	1.5189	0.078*	
C2	0.2027 (5)	−0.2183 (5)	1.4156 (4)	0.0628 (10)	
H2B	0.1794	−0.2530	1.4733	0.075*	
C3	0.1376 (6)	−0.2861 (5)	1.3011 (4)	0.0655 (11)	
H3A	0.0698	−0.3679	1.2799	0.079*	
C4	0.1749 (5)	−0.2300 (4)	1.2179 (4)	0.0562 (9)	
H4A	0.1348	−0.2746	1.1397	0.067*	
C5	0.2727 (4)	−0.1070 (3)	1.2534 (3)	0.0417 (7)	
C6	0.3068 (4)	−0.0356 (3)	1.1717 (3)	0.0375 (6)	
C7	0.2136 (4)	−0.0771 (3)	1.0636 (3)	0.0377 (6)	
H7A	0.1342	−0.1533	1.0386	0.045*	
C8	0.2430 (3)	−0.0020 (3)	0.9955 (2)	0.0344 (6)	
C9	0.1483 (3)	−0.0380 (3)	0.8802 (2)	0.0358 (6)	
C10	0.0349 (4)	−0.1544 (3)	0.8286 (3)	0.0460 (7)	
H10A	0.0137	−0.2141	0.8658	0.055*	
C11	−0.0458 (5)	−0.1798 (4)	0.7204 (3)	0.0538 (9)	
H11A	−0.1233	−0.2562	0.6850	0.065*	
C12	−0.0105 (4)	−0.0919 (4)	0.6666 (3)	0.0526 (8)	
H12A	−0.0620	−0.1085	0.5934	0.063*	
C13	0.1019 (4)	0.0213 (4)	0.7218 (3)	0.0483 (8)	
H13A	0.1242	0.0815	0.6852	0.058*	
C14	0.4514 (3)	0.1485 (3)	1.1287 (2)	0.0354 (6)	
C15	0.5791 (4)	0.2644 (3)	1.1610 (3)	0.0385 (6)	
C16	0.6641 (4)	0.3068 (3)	1.2730 (3)	0.0448 (7)	
C17	0.6313 (4)	0.3365 (3)	1.0886 (3)	0.0392 (6)	
C18	0.4729 (4)	0.3546 (4)	0.7681 (3)	0.0460 (7)	
C19	0.3704 (7)	0.3062 (5)	0.5696 (4)	0.0831 (15)	
H19A	0.2953	0.2405	0.5858	0.125*	
H19B	0.4275	0.2655	0.5053	0.125*	
H19C	0.3140	0.3627	0.5496	0.125*	
C20	0.6072 (6)	0.4868 (4)	0.6641 (4)	0.0608 (10)	
H20A	0.6742	0.5285	0.7370	0.091*	
H20B	0.5583	0.5493	0.6478	0.091*	
H20C	0.6709	0.4516	0.6022	0.091*	
H1	0.546 (5)	0.406 (4)	0.831 (4)	0.056 (11)*	

### Atomic displacement parameters (Å^2^)

**Table:**

	*U*^11^	*U*^22^	*U*^33^	*U*^12^	*U*^13^	*U*^23^
Zn1	0.0394 (2)	0.0427 (2)	0.0365 (2)	0.00096 (15)	0.00087 (14)	0.02205 (16)
Cl1	0.0495 (5)	0.0453 (4)	0.0573 (5)	0.0067 (3)	0.0090 (4)	0.0242 (4)
O1	0.0415 (12)	0.0654 (16)	0.0501 (13)	−0.0046 (11)	−0.0003 (10)	0.0344 (12)
O2	0.0582 (15)	0.0631 (16)	0.0436 (13)	−0.0035 (12)	0.0051 (11)	0.0313 (12)
N1	0.068 (2)	0.0629 (19)	0.0421 (15)	0.0026 (15)	−0.0026 (14)	0.0297 (14)
N2	0.0516 (16)	0.0552 (17)	0.0468 (15)	0.0107 (13)	0.0047 (12)	0.0251 (13)
N3	0.0365 (12)	0.0333 (11)	0.0290 (11)	0.0053 (9)	0.0033 (9)	0.0130 (9)
N4	0.0424 (13)	0.0445 (14)	0.0350 (13)	0.0048 (11)	0.0015 (10)	0.0191 (11)
N5	0.074 (2)	0.079 (3)	0.055 (2)	−0.0010 (19)	−0.0158 (17)	0.0238 (18)
N6	0.0483 (17)	0.0606 (19)	0.067 (2)	−0.0140 (14)	−0.0101 (14)	0.0364 (16)
N7	0.0607 (18)	0.0525 (16)	0.0411 (14)	0.0061 (13)	0.0101 (13)	0.0257 (12)
C1	0.079 (3)	0.077 (3)	0.046 (2)	0.009 (2)	0.0025 (18)	0.0359 (19)
C2	0.068 (2)	0.080 (3)	0.063 (2)	0.018 (2)	0.0154 (19)	0.052 (2)
C3	0.070 (3)	0.063 (2)	0.073 (3)	−0.003 (2)	0.000 (2)	0.046 (2)
C4	0.063 (2)	0.056 (2)	0.055 (2)	−0.0007 (17)	−0.0042 (17)	0.0341 (17)
C5	0.0447 (16)	0.0456 (17)	0.0432 (16)	0.0119 (13)	0.0056 (12)	0.0253 (13)
C6	0.0407 (15)	0.0404 (15)	0.0376 (15)	0.0112 (12)	0.0084 (12)	0.0202 (12)
C7	0.0402 (15)	0.0362 (14)	0.0373 (14)	0.0037 (11)	0.0035 (11)	0.0169 (12)
C8	0.0369 (14)	0.0351 (14)	0.0325 (13)	0.0080 (11)	0.0044 (11)	0.0137 (11)
C9	0.0342 (14)	0.0401 (15)	0.0333 (14)	0.0081 (11)	0.0044 (11)	0.0138 (11)
C10	0.0490 (18)	0.0418 (16)	0.0439 (17)	0.0021 (13)	−0.0014 (13)	0.0164 (13)
C11	0.0513 (19)	0.0502 (19)	0.0464 (18)	−0.0023 (15)	−0.0069 (15)	0.0092 (15)
C12	0.0515 (19)	0.062 (2)	0.0381 (17)	0.0084 (16)	−0.0079 (14)	0.0143 (15)
C13	0.0515 (19)	0.057 (2)	0.0364 (16)	0.0049 (15)	−0.0040 (13)	0.0217 (14)
C14	0.0358 (14)	0.0375 (14)	0.0354 (14)	0.0085 (11)	0.0046 (11)	0.0161 (11)
C15	0.0376 (14)	0.0402 (15)	0.0376 (15)	0.0041 (12)	0.0004 (11)	0.0164 (12)
C16	0.0436 (17)	0.0468 (17)	0.0401 (17)	0.0031 (13)	−0.0038 (13)	0.0153 (13)
C17	0.0332 (14)	0.0401 (15)	0.0434 (16)	0.0045 (11)	0.0018 (11)	0.0163 (13)
C18	0.0488 (18)	0.0525 (19)	0.0398 (16)	0.0079 (15)	0.0064 (13)	0.0223 (14)
C19	0.110 (4)	0.086 (3)	0.048 (2)	−0.009 (3)	−0.012 (2)	0.036 (2)
C20	0.075 (3)	0.058 (2)	0.061 (2)	0.0138 (19)	0.027 (2)	0.0358 (18)

### Geometric parameters (Å, °)

**Table:**

Zn1—O1	1.996 (3)	C3—H3A	0.9300
Zn1—O2	2.066 (2)	C4—C5	1.380 (5)
Zn1—N4	2.103 (3)	C4—H4A	0.9300
Zn1—N3	2.106 (2)	C5—C6	1.491 (4)
Zn1—Cl1	2.2956 (12)	C6—C7	1.406 (4)
O1—C17	1.256 (4)	C7—C8	1.378 (4)
O2—C18	1.234 (4)	C7—H7A	0.9300
N1—C1	1.338 (5)	C8—C9	1.493 (4)
N1—C5	1.339 (5)	C9—C10	1.394 (4)
N2—C6	1.378 (4)	C10—C11	1.388 (5)
N2—C14	1.416 (4)	C10—H10A	0.9300
N3—C8	1.350 (4)	C11—C12	1.362 (6)
N3—C14	1.350 (4)	C11—H11A	0.9300
N4—C9	1.339 (4)	C12—C13	1.372 (5)
N4—C13	1.353 (4)	C12—H12A	0.9300
N5—C16	1.136 (5)	C13—H13A	0.9300
N6—C17	1.355 (4)	C14—C15	1.451 (4)
N6—H6A	0.8600	C15—C16	1.413 (4)
N6—H6B	0.8600	C15—C17	1.426 (4)
N7—C18	1.314 (4)	C18—H1	0.91 (4)
N7—C19	1.444 (6)	C19—H19A	0.9600
N7—C20	1.465 (5)	C19—H19B	0.9600
C1—C2	1.357 (6)	C19—H19C	0.9600
C1—H1B	0.9300	C20—H20A	0.9600
C2—C3	1.379 (6)	C20—H20B	0.9600
C2—H2B	0.9300	C20—H20C	0.9600
C3—C4	1.389 (5)		
			
Cg1···Cg1^i^	3.809 (3)	Cg1···Cg2^ii^	3.834 (3)
			
O1—Zn1—O2	89.37 (11)	C8—C7—H7A	120.9
O1—Zn1—N4	146.12 (12)	C6—C7—H7A	120.9
O2—Zn1—N4	88.67 (11)	N3—C8—C7	123.4 (3)
O1—Zn1—N3	87.29 (10)	N3—C8—C9	114.8 (3)
O2—Zn1—N3	149.32 (11)	C7—C8—C9	121.8 (3)
N4—Zn1—N3	77.58 (10)	N4—C9—C10	121.5 (3)
O1—Zn1—Cl1	107.51 (9)	N4—C9—C8	115.4 (3)
O2—Zn1—Cl1	103.78 (9)	C10—C9—C8	123.1 (3)
N4—Zn1—Cl1	105.79 (8)	C11—C10—C9	118.8 (3)
N3—Zn1—Cl1	106.30 (7)	C11—C10—H10A	120.6
C17—O1—Zn1	126.9 (2)	C9—C10—H10A	120.6
C18—O2—Zn1	125.8 (2)	C12—C11—C10	119.5 (3)
C1—N1—C5	117.3 (3)	C12—C11—H11A	120.3
C6—N2—C14	120.6 (3)	C10—C11—H11A	120.3
C8—N3—C14	119.3 (2)	C11—C12—C13	119.2 (3)
C8—N3—Zn1	114.61 (19)	C11—C12—H12A	120.4
C14—N3—Zn1	124.8 (2)	C13—C12—H12A	120.4
C9—N4—C13	118.6 (3)	N4—C13—C12	122.5 (3)
C9—N4—Zn1	115.2 (2)	N4—C13—H13A	118.8
C13—N4—Zn1	125.8 (2)	C12—C13—H13A	118.8
C17—N6—H6A	120.0	N3—C14—N2	119.8 (3)
C17—N6—H6B	120.0	N3—C14—C15	119.9 (3)
H6A—N6—H6B	120.0	N2—C14—C15	120.3 (3)
C18—N7—C19	120.9 (3)	C16—C15—C17	116.8 (3)
C18—N7—C20	121.7 (3)	C16—C15—C14	117.0 (3)
C19—N7—C20	117.4 (3)	C17—C15—C14	126.1 (3)
N1—C1—C2	124.4 (4)	N5—C16—C15	177.3 (4)
N1—C1—H1B	117.8	O1—C17—N6	115.8 (3)
C2—C1—H1B	117.8	O1—C17—C15	125.4 (3)
C1—C2—C3	118.3 (4)	N6—C17—C15	118.9 (3)
C1—C2—H2B	120.9	O2—C18—N7	123.6 (3)
C3—C2—H2B	120.9	O2—C18—H1	119 (3)
C2—C3—C4	118.8 (4)	N7—C18—H1	117 (3)
C2—C3—H3A	120.6	N7—C19—H19A	109.5
C4—C3—H3A	120.6	N7—C19—H19B	109.5
C5—C4—C3	118.8 (4)	H19A—C19—H19B	109.5
C5—C4—H4A	120.6	N7—C19—H19C	109.5
C3—C4—H4A	120.6	H19A—C19—H19C	109.5
N1—C5—C4	122.3 (3)	H19B—C19—H19C	109.5
N1—C5—C6	115.6 (3)	N7—C20—H20A	109.5
C4—C5—C6	122.0 (3)	N7—C20—H20B	109.5
N2—C6—C7	118.5 (3)	H20A—C20—H20B	109.5
N2—C6—C5	120.6 (3)	N7—C20—H20C	109.5
C7—C6—C5	120.8 (3)	H20A—C20—H20C	109.5
C8—C7—C6	118.3 (3)	H20B—C20—H20C	109.5

Symmetry codes: (i) −*x*+1, −*y*+2, −*z*; (ii) −*x*+2, −*y*+2, −*z*.

### Hydrogen-bond geometry (Å, °)

**Table:**

*D*—H···*A*	*D*—H	H···*A*	*D*···*A*	*D*—H···*A*
N6—H6A···Cl1^iii^	0.86	2.57	3.396 (4)	161
C10—H10A···Cl1^iv^	0.93	2.80	3.670 (4)	156

Symmetry codes: (iii) −*x*+1, −*y*+1, −*z*+2; (iv) −*x*, −*y*, −*z*+2.
